# Circulating PCSK9 and cardiovascular events in FH patients with standard lipid-lowering therapy

**DOI:** 10.1186/s12967-019-2123-9

**Published:** 2019-11-11

**Authors:** Ye-Xuan Cao, Jing-Lu Jin, Di Sun, Hui-Hui Liu, Yuan-Lin Guo, Na-Qiong Wu, Rui-Xia Xu, Cheng-Gang Zhu, Qian Dong, Jing Sun, Jian-Jun Li

**Affiliations:** Division of Dyslipidemia, State Key Laboratory of Cardiovascular Disease, Fu Wai Hospital, National Center for Cardiovascular Diseases, Chinese Academy of Medical Sciences, Peking Union Medical College, BeiLiShi Road 167, Beijing, 100037 China

**Keywords:** PCSK9, Familial hypercholesterolemia, Prospective cohort

## Abstract

**Background:**

Proprotein convertase subtilisin/kexin 9 (PCSK9) has been proposed as a novel target for coronary artery disease (CAD). Familial hypercholesterolemia (FH) is characterized by high prevalence of CAD and major cardiovascular events (MACEs). However, no data is available on the association between PCSK9 levels and MACEs in FH patients with standard lipid lowering therapy.

**Methods:**

A total of 338 consecutive heterozygous FH (Dutch Lipid Clinic Network score ≥ 6) was enrolled and followed up for the occurrence of MACEs. Multidetector CT and coronary angiography were performed to determine coronary artery calcification score (CACS) and Gensini score (GS). Multivariable Cox regression analyses were used to calculate hazard ratios (HRs) with 95% confidence intervals (CIs). Plasma PCSK9 concentrations were determined by enzyme immunoassay.

**Results:**

PCSK9 was independently and positively associated CACS and GS at baseline. During a mean follow-up of 3 years, 33 (9.8%) events occurred. Patients with MACEs had higher median PCSK9 compared with those without (332.47 vs. 311.89 ng/mL, p = 0.038). Kaplan–Meier analysis revealed that patients with higher PCSK9 presented lower event-free survival (p = 0.0017). PCSK9 was statistically correlated with MACEs after adjusting for confounding factors, with the HR per SD being 1.86 (1.31–2.65) and 3.70 (1.16–11.82) for the highest tertile compared with the lowest tertile. Adding PCSK9 to Cox prediction model led to a statistical improvement in net reclassification and integrated discrimination.

**Conclusion:**

Elevated levels of PCSK9 were positively associated with the development of CAD and future cardiovascular events, suggesting that measurement of PCSK9 concentration might be useful for cardiovascular risk stratification. Further studies are needed to confirm our results.

## Background

Proprotein convertase substilisin/kexin type 9 (PCSK9), a serine protease mainly expressed in the liver, is mostly known for its role in the regulation of cholesterol metabolism [1]. PCSK9 binds hepatic low-density lipoprotein receptor (LDLR) and promotes its degradation, which results in increased plasma low-density lipoprotein cholesterol (LDL-C) concentrations and higher risk of coronary artery disease (CAD) [2]. Gain-of-function mutations in *PCSK9* gene causing over-expression of the plasma PCSK9 are associated with familial hypercholesterolemia (FH) [3]. Consequently, human PCSK9 monoclonal antibodies have been developed as a novel lipid-lowering strategy. Mounting randomized controlled trials and meta-analysis suggested that PCSK9 antibodies could significantly reduce more than 50% circulating LDL-C levels and decrease future cardiovascular events across various dyslipidemic populations [4–6].

Although most studies investigated the effects of PCSK9 on LDL-C metabolism, an increasing number of experimental and clinical studies have demonstrated that PCSK9 contributed directly to the progression of atherosclerosis by enhancing the expression of pro-inflammatory genes, promoting apoptotic cell death and leading to endothelial dysfunction independent of its effect on the LDLR [7, 8]. In this situation, the plasma concentration of PCSK9 has attracted scientific interest as a novel marker for major adverse cardiovascular events (MACEs). Previous studies have examined the relationship between PCSK9 concentrations and cardiovascular outcomes in general population, but with available divergent results [9–12]. It is noticeable that the association between plasma concentration of PCSK9 and future MACEs has not been investigated in the setting of FH.

FH is an autosomal co-dominant disorder caused by defects in *LDLR*, apolipoprotein B (*APOB*) and *PCSK9* genes [13, 14]. The pathological alterations lead to extremely raised LDL-C levels and increased risk for premature CAD [15, 16]. Early diagnosis and an aggressive cholesterol-lowering treatment could prevent the occurrence of cardiovascular events by reducing the long-term exposure of LDL-C and improve quality of life [15, 17]. Although FH patients are considered as a high CAD risk group, not all these patients with elevated LDL-C levels will develop MACEs, indicating that the realistic risk is heterogeneous [18]. Additionally, it has been shown that FH patients with high circulating PCSK9 levels tend to present with worse hypercholesterolemic phenotype [19]. Therefore, founding a novel marker for further stratification of the cardiovascular risk for FH patients is of great interest.

Given the importance of PCSK9 as a potential atherogenic risk marker and the undetermined relationship between circulating PCSK9 and incident MACEs in FH patients, in the present study, we sought to evaluate the prognostic value of plasma PCSK9 levels and its ability to reclassify risk among patients with FH in a Chinese FH prospective cohort.

## Methods

### Study design and population

The study protocol was approved by the hospital’s ethical review board (Fu Wai Hospital and National Center for Cardiovascular Diseases, Beijing, China) and all patients enrolled in the study provided written informed consent. This study was conducted in line with the declaration of Helsinki.

A total of 60,064 consecutive patients who were hospitalized in Fuwai Hospital because of angina-like chest pain and/or hyperlipidaemia from April 2011 to November 2018 were collected. Among them, all FH patients were selected for the present study regardless of whether they had pre-existing CAD or not before enrolment. Clinical FH diagnosis was established by Dutch Lipid Clinic Network (DLCN) criteria and only patients with a DLCN score ≥ 6 were included (definite and probable; Additional file 1: Table S1). If the untreated lipid profiles were unavailable during the diagnosis, we calculated the untreated LDL-C levels by a correction factor depending on the type and potency of lipid-lowering drugs for the participants receiving lipid-lowering medications [20]. Participants were excluded from the study if they aged < 18, had a history of severe chronic cardiac failure, thyroid dysfunction, severe liver and/or renal insufficiency, malignant disease or were homozygous FH patients. All the HeFH patients received standard statin therapy which was defined as rosuvastatin dose 20 mg plus ezetimibe 10 mg.

### Baseline data collection and genetic analysis

In a baseline survey, participants completed a detailed questionnaire including medical history, family history and lifestyle and visited a clinic. Clinical data, including age, height, weight, blood pressure and heart beat were obtained by experienced physicians and nurses. BMI was calculated by the square of weight divided by height. Hypertension was defined as systolic BP ≥ 140 mmHg or diastolic BP ≥ 90 mmHg or the use of antihypertensive medication. Diabetes was considered present if 1 of 4 criteria was met: self-reported diabetes, fasting blood glucose level ≥ 7.0 mmol/L (126 mg/dL), fasting plasma glycemic levels ≥ 126 mg/dL, or use of antidiabetic medication or insulin injections. Alcohol drinkers referred to regular drinking within the previous 3 months.

Blood samples for laboratory examinations were obtained from the cubital vein after fasting for at least 12 h upon admission. The concentrations of plasma total cholesterol (TC), triglyceride (TG), LDL-C, high-density lipoprotein cholesterol (HDL-C), apolipoprotein A (apoA) and apoB were measured using an automatic biochemistry analyzer (Hitachi 7150, Japan), while Lp(a) levels were assayed by an immunoturbidimetry method. The concentration of high sensitive C-reactive protein was measured by immunoturbidimetry (Beckmann Assay360, Bera, California) as previously described [10]. Other related biochemical and hematological indicators were measured according to standard tests. Plasma PCSK9 concentrations were determined by a commercial sandwich enzyme immunoassay (Quantikine ELISA, R&D System Europe Ltd, Minneapolis, USA) with a lower limit of detection of 0.096 ng/mL. The intra-assay and inter-assay coefficients of variation were < 5% as previous described [21, 22].

Genetic testing was performed to test *LDLR/APOB/PCSK9* pathogenic variants as previously described [17]. Double heterozygote was defined as two pathogenic variants in the same gene and compound heterozygote was defined as pathogenic variants in *LDLR* and *APOB* [23].

### CACS and Gensini score

A 64-slice scanner (Light Speed VCT, GE Healthcare, Milwaukee, Wisconsin) with a rotation time of 0.35-s and a pitch of 0.16 to 0.22 was used to obtain coronary artery calcification score (CACS) [24]. The machine was setup with the tube current of 200 to 550 mA and tube voltage of 120 kV, and image acquisition was performed during diastole. All patients should have heart rates ≤ 70 beats/min when undergoing this procedure, otherwise beta-blockers would be given to individuals without contraindication orally 1 h before scanning. All data sets were reconstructed using retrospective electrocardiography (ECG)-gated sequential scan during 40% to 80% RR interval. Data sets were used to a dedicated workstation (Deep Blue, ADW4.3, GE Healthcare). At least 3 contiguous pixels present, and a CT threshold of 130 HU was defined as calcium. The CACS of each lesion was calculated by multiplying lesion area by a density factor as developed by Agatston et al. [25]. Furthermore, CAC severity was categorized into three groups according to scores of 0, 0–100 and > 100.

Coronary angiography was performed according to standard Judkins techniques by two experienced independent interventional physicians who were blinded to patients’ clinical characteristics. The presence of coronary stenosis with luminal diameter narrowing of 50% or more in any major coronary vessels was considered as CAD. The severity of coronary atherosclerosis was evaluated by the Gensini system [26]. More specifically, stenosis of 25%, 50%, 75%, 90%, 99% and 100% were given score of 1, 2, 4, 8, 16 and 32, respectively. Segment weighting factors are between 0.5 and 5.0. Finally, the Gensini score was calculated by summation of all segment scores (each segment score equals a segment weighting factor multiplied by a severity score). Scoring was performed by two experienced investigators and averaged.

### Outcome assessment

Patients were followed up every half-year by telephone, E-mail and clinic revisit conducted by trained nurses or doctors, who were blinded to the information of patients up to the last day of the follow-up period or until death occurred. The composite clinical endpoint included the following major adverse cardiovascular events (MACEs): myocardial infarction (MI), unstable angina requiring readmission, ischemic stroke, coronary revascularization and cardiovascular death (Additional file 1: Table S2). Endpoint of MI was confirmed if review of medical records showed diagnostic symptom patterns, typical electrocardiogram changes and elevated myocardial enzyme. Diagnosis of unstable angina was on the basis of rest angina or new-onset severe angina with normal serum levels of cardiac enzymes that required hospitalization. Ischemic stroke was described as acute cerebral infarction symptoms lasting more than 24 h with diagnostic CT or MRI. Coronary revascularization was defined as percutaneous coronary intervention (PCI) and coronary artery bypass grafting (CABG) later than 90 days after discharge. Diagnosis of cardiovascular death was confirmed with hospital records, death certificates and information provided by family members. Hard endpoints were defined as MI, ischemic stroke and cardiovascular death. During the follow-up period, 4 patients (1.2%) were lost to follow-up. Finally, a total of 338 HeFH patients were included in the current study. At the end of follow-up, 17 (5.0%) cases did not use statin (7 because of statin intolerance and 10 because of poor adherence to treatment).

### Statistical analyses

All statistical analyses were performed using SPSS version 20.0 software (SPSS Inc., Chicago, IL, USA) and R version 3.2.2 (R Foundation for Statistical Computing, Vienna, Austria). The results are presented as mean ± standard deviation (SD) or medians (interquartile range, IQR) for continuous variables and as number (%) for categorical variables. Student’s *t* test, the Mann–Whitney U-test, χ2 analysis and Fisher’s test and ANOVA analysis were used to compare the statistical differences between groups as appropriate. Bonferroni correction was performed for multiple testing and p < 0.017 was needed to assert statistical significance (0.05/3 = 0.017). Spearman correlation coefficients were calculated to evaluate the correlation between parameters. Univariate and multivariate linear regression analyses were performed to investigate the associations of PCSK9, CACS and Gensini score. The event-free survival rates among tertiles of PCSK9 were estimated by the Kaplan–Meier method and compared by the log-rank test. Using the Cox regression analyses, hazard ratios (HRs) of incident MACE with 95% confidence intervals (CIs) were calculated. In the multivariable Cox model, we adjusted for known cardiovascular risk factors and confounders with significant association observed at univariate analysis. PCSK9 was examined as a continuous variable (per SD) but also by tertiles to obtain robust associations. Age- and sex-adjusted restricted cubic spline was used to assess the potential linear relationship between PCSK9 levels with MACEs. We also performed post hoc sensitivity analysis of the potential influence of other individual predictors on the association between PCSK9 and events which were forced into multivariate models together with PCSK9. We performed several subgroup analyses to test the robustness of our findings. To analyze the incremental value of adding continuous PCSK9 variable to risk prediction model [including age, male, smoking, diabetes, LDL-C, HDL-C and Lp(a)], we calculated receiver operating characteristic curve, Harrell’s C-statistic, continuous net reclassification improvement (NRI) and integrated discrimination improvement (IDI).

## Results

### Baseline characteristics

During the follow-up period, 338 HeFH patients were consecutively enrolled in the present study. The population consisted of 198 (58.6%) men and 140 (41.4%) women. The median age was 49.38 ± 11.35 years. The interquartile PCSK9 levels ranged from 249.97 to 396.24 ng/mL with a median of 322.18 ng/mL. Table 1 displays patient characteristics of the study cohort at baseline and according to event occurrence during follow-up. Patients with events showed clinical features as higher levels of Lp(a) [55.09 (26.96–77.82) vs 32.29 (13.37–66.19), p = 0.025] and higher percentage of hypertension (63.6% vs 41.0%, p = 0.012). However, no difference was revealed in the levels of TG, TC and LDL-C. Besides, there was no significant difference between the two groups in currently smoking, diabetes, baseline statin use and tendon xanthoma (all p > 0.05). In addition, concentration of PCSK9 was statistically higher in events group [332.47 (294.74–448.96) vs 311.89 (246.73–386.61), p = 0.038; Additional file 1: Fig. S1].

**Table 1:** Clinical and laboratory characteristics of FH patients with or without an incident cardiovascular event

Variables	Overall (N = 338)	Without events (N = 305)	With events (N = 33)	p-value
*Clinical factors*				
Age, years	49.38 ± 11.35	49.34 ± 11.32	49.67 ± 11.81	0.877
Male, n (%)	198 (58.6)	178 (58.4)	20 (60.6)	0.894
BMI, kg/(m^2^)	25.49 ± 3.41	25.42 ± 3.38	26.11 ± 3.66	0.282
Normal weight, n (%)	155 (45.9)	143 (46.9)	12 (36.4)	
Overweight, n (%)	134 (39.6)	119 (39.0)	15 (45.5)	
Obesity, n (%)	49 (14.5)	43 (14.1)	6 (18.2)	
Prior CAD, n (%)	189 (55.9)	166 (54.4)	23 (69.7)	0.093
Family history of CAD, n (%)	157 (46.4)	145 (47.5)	12 (36.4)	0.221
Currently smoking, n (%)	141 (41.7)	126 (41.3)	15 (45.5)	0.657
Alcohol drinker, n (%)	67 (19.8)	61 (20.0)	6 (18.2)	0.173
Hypertension, n (%)	146 (43.2)	125 (41.0)	21 (63.6)	0.012
Diabetes, n (%)	61 (18.0)	55 (18.0)	6 (18.2)	0.971
Baseline statin use, n (%)	263 (77.8)	234 (76.7)	29 (87.9)	0.143
Tendon xanthoma, n (%)	26 (7.7)	24 (7.9)	2 (6.1)	0.711
Definite FH, n (%)	222 (65.7)	199 (65.2)	23 (69.7)	0.609
*Laboratory factors*				
TG, mmol/L	1.63 (1.21–2.14)	1.61 (1.18–2.11)	1.84 (1.40–2.46)	0.210
TC, mmol/L	7.00 ± 2.07	6.98 ± 2.03	7.24 ± 2.41	0.488
HDL-C, mmol/L	1.11 ± 0.32	1.12 ± 0.32	1.02 ± 0.33	0.110
LDL-C, mmol/L	5.20 ± 1.78	5.19 ± 1.79	5.23 ± 1.75	0.901
ApoA, g/L	1.31 ± 0.31	1.32 ± 0.31	1.28 ± 0.37	0.502
ApoB, g/L	1.45 ± 0.45	1.44 ± 0.45	1.56 ± 0.47	0.141
Lp(a), mg/dL	34.95 (14.35–67.11)	32.29 (13.37–66.19)	55.09 (26.96–77.82)	0.025
HsCRP, mg/L	1.52 (0.71–3.20)	1.40 (0.69–3.06)	2.34 (0.88–4.67)	0.095
FPG, mmol/L	5.54 ± 1.50	5.48 ± 1.44	6.02 ± 1.87	0.115
HbA1C, %	6.12 ± 1.05	6.10 ± 1.02	6.28 ± 1.27	0.364
WBC, ng/mL	6.34 ± 1.78	6.31 ± 1.81	6.54 ± 1.52	0.475
LYM, ng/mL	2.06 ± 0.66	2.07 ± 0.68	1.92 ± 0.50	0.226
PCSK9, ng/mL	322.18 (249.97–396.24)	311.89 (246.73–386.61)	332.47 (294.74–448.96)	0.038
FH mutations	191 (56.5)	172 (57.0)	19 (56.4)	0.896
*LDLR*, n (%)	126 (37.3)	116 (38.0)	10 (30.3)	
*APOB*, n (%)	31 (9.2)	24 (7.9)	7 (21.2)	
*PCSK9*, n (%)	5 (0.9)	2 (0.7)	1 (3.0)	
Double/Compound heterozygote, n (%)	31 (9.2)	29 (9.5)	2 (6.1)	

Data are expressed as mean ± standard deviation, median (interquartile range) or n (%)

*FH* familial hypercholesterolemia, *BMI* body mass index, *CAD* coronary artery disease, *TG* triglyceride, *TC* total cholesterol, *HDL-C* high-density lipoprotein cholesterol, *LDL-C* low-density lipoprotein cholesterol, *ApoA* apolipoprotein A, *ApoB* apolipoprotein B, *Lp(a)* lipoprotein(a), *hsCRP* high sensitivity C-reactive protein, *FPG* fasting plasma glucose, *HbA1c* glycosylated hemoglobin, *WBC* white blood cell, *LYM* lymphocyte, *PCSK9* proprotein convertase subtilisin-kexin type 9, *LDLR* low-density lipoprotein cholesterol receptor

The entire study population was also categorized by tertiles of the plasma PCSK9 concentrations at baseline (Table 2). FH patients in high PCSK9 group had a significantly higher occurrence of tendon xanthoma and higher levels of TC, LDL-C, ApoB and Lp(a) (all p < 0.05). Participants with higher levels of PCSK9 were also more likely to be alcohol drinker (p = 0.008). There was no difference in age, BMI, smoking status, hypertension, diabetes and baseline statin use.

**Table 2:** Clinical and laboratory characteristics of FH patients at baseline stratified by tertile of baseline PCSK9 concentration

Variables	PCSK9 concentration tertile			
	Tertile 1 (< 274 ng/mL)	Tertile 2 (274–369 ng/mL)	Tertile 3 (> 369 ng/mL)	p-value for trend
*Clinical factors*				
Age, years	49.11 ± 9.85	50.86 ± 12.30	48.16± 11.69	0.193
Male, n (%)	78 (69.6)	59 (52.2)	61 (54.0)	0.014
BMI, kg/(m^2^)	25.70 ± 3.55	25.74 ± 3.01	25.04 ± 3.62	0.267
Prior CAD, n (%)	51 (45.5)	61 (54.0)	77 (68.1)	0.003
Family history of CAD, n (%)	52 (46.4)	55 (48.7)	50 (44.2)	0.801
Currently smoking, n (%)	49 (43.8)	34 (30.1)	43 (38.1)	0.178
Alcohol drinker, n (%)	30 (26.8)	31 (27.4)	14 (12.4)	0.008
Hypertension, n (%)	45 (40.2)	47 (41.6)	42 (37.2)	0.747
Diabetes, n (%)	23 (20.5)	17 (15.0)	16 (14.2)	0.422
Baseline statin use, n (%)	92 (82.1)	84 (74.3)	87 (77.0)	0.359
Tendon xanthoma	3 (2.7)	5 (4.4)	18 (15.9)	<0.001
*Laboratory factors*				
TG, mmol/L	1.70 (1.34–2.20)	1.63 (1.11–2.03)	1.53 (1.15–2.12)	0.146
TC, mmol/L	6.41 ± 1.59	6.97 ± 2.04	7.62 ± 2.35	0.001
HDL-C, mmol/L	1.07 ± 0.33	1.19 ± 0.31	1.08 ± 0.30	0.005
LDL-C, mmol/L	4.68 ± 1.46	5.11 ± 1.56	5.80 ± 2.09	0.001
ApoA, g/L	1.27 ± 0.30	1.41 ± 0.30	1.25 ± 0.32	0.001
ApoB, g/L	1.31 ± 0.34	1.42 ± 0.40	1.61 ± 0.54	0.001
Lp(a), mg/dL	20.81 (9.71–48.41)	33.86 (14.73–66.05)	46.60 (24.87–81.78)	0.001
hsCRP, mg/L	1.58 (0.76–3.55)	1.41 (0.702.79)	1.58 (0.71–3.42)	0.654
FPG, mmol/L	5.62 ± 1.76	5.49 ± 1.39	5.51 ± 1.32	0.787
HbA1C, %	6.21 ± 1.22	6.09 ± 0.98	6.05 ± 0.92	0.525
WBC, ng/mL	6.30 ± 1.79	6.21 ± 1.57	6.48 ± 1.96	0.556
LYM, ng/mL	2.04± 0.70	2.09± 0.62	2.04 ± 0.67	0.832

Data are expressed as mean ± standard deviation, median (interquartile range) or n (%)

*FH* familial hypercholesterolemia, *BMI* body mass index, *CAD* coronary artery disease, *TG* triglyceride, *TC* total cholesterol, *HDL-C* high-density lipoprotein cholesterol, *LDL-C* low-density lipoprotein cholesterol, *ApoA* apolipoprotein A, *ApoB* apolipoprotein B, *Lp(a)* lipoprotein(a), *hsCRP* high sensitivity C-reactive protein, *FPG* fasting plasma glucose, *HbA1c* glycosylated hemoglobin, *WBC* white blood cell, *LYM* lymphocyte

### Association of PCSK9 level with baseline characteristics

Correlations between plasma PCSK9 levels and demographic, clinic and laboratory characteristics are shown in Additional file 1: Table S3 and Fig. 2. By applying spearman correlation analyses, we found that PCSK9 concentration was positively correlated with male sex and tendon xanthoma. Likewise, plasma PCSK9 was found to be positively correlated with TC, LDL-C, ApoB and Lp(a). PCSK9 concentration was not significantly related to smoking, hypertension or diabetes.

### Correlations of coronary atherosclerosis with PCSK9

Patient clinical characteristics according to CACS and coronary severity are reported in Additional file 1: Tables S4 and S5. Overall, 28 patients (17.2%) had CAC scores of 0, whereas 71 (43.8%) had CAC scores of 1 to 100 and 63 (38.9%) had CAC scores > 100. Highest baseline PCSK9 levels were seen in those with CAC scores > 100 (p < 0.001, Additional file 1: Fig. S3). Furthermore, the levels of PCSK9 were significantly higher in the upper tertile of Gensini score. In the correlation analyses, PCSK9 level was found to be positively associated with CACS (r = 0.504, p = 0.001) and Gensini score (r = 0.426, p = 0.022; Additional file 1: Table S6). Multivariable analysis showed that PCSK9 level was independently associated with CACS in FH patients (β = 0.449, p < 0.001; Additional file 1: Table S7). A similar positive association between plasma PCSK9 concentration and Gensini score was also found after adjustment with confounding factors (β = 0.435, p = 0.014).

### Cardiovascular outcomes during follow-up

On follow-up (median 36 months, interquartile range: 19–51 month), 33 FH participants (9.8%) developed a MACE. Among them, 9 (27.3%) died, 2 (6.1%) developed MI, 6 (18.2%) had stroke, 4 (12.1%) experienced readmission due to unstable angina pectoris and 12 (36.4%) underwent PCI or CABG. Event rate increased progressively from 5.4% in patients with lowest concentration of PCSK9 to 15.4% of the patients with highest PCSK9 concentrations. The events per 1,000 person-years were 32.5 (95%CI 22.7–45.2) in the overall group, and likewise elevated progressively with increasing PCSK9 levels: 12.8 in the first tertile, 31.6 in median tertile and 58.2 in the highest tertile. The difference between Tertile 3 and Tertile 1 achieved Bonferroni-adjusted significance (p = 0.012).

**Fig. 1:**
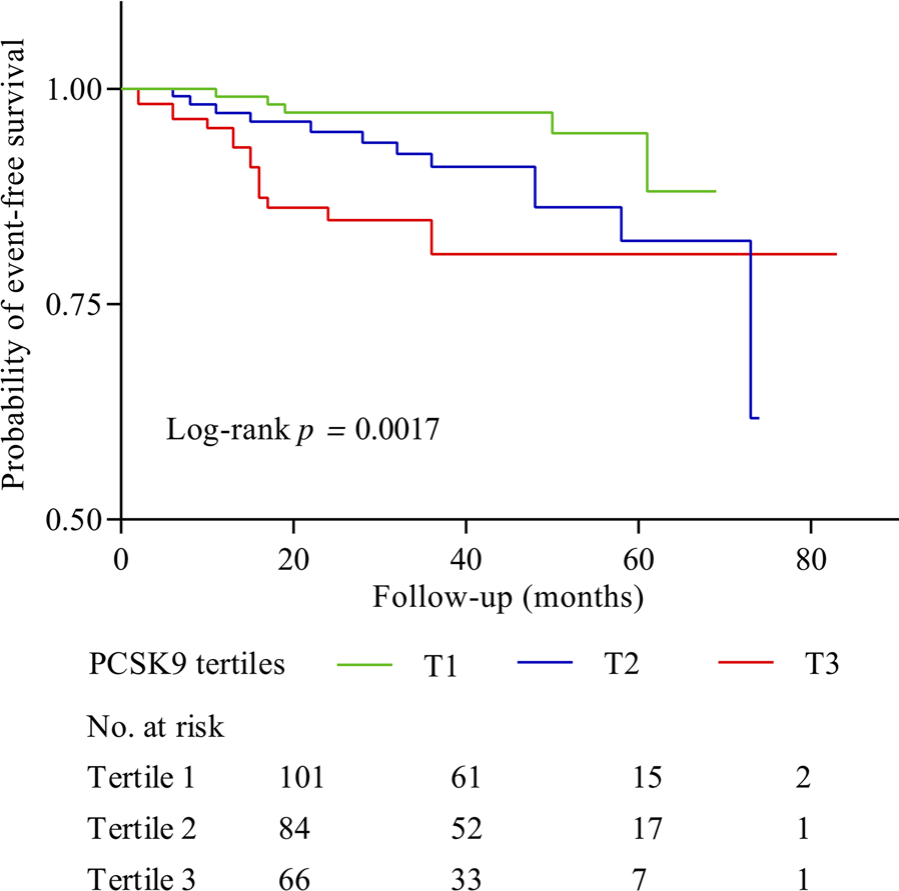
Kaplan-Meier curves of the cumulative incidence of MACE according to PCSK9 tertiles at baseline. The log-rank test was used to compare event-free survival among PCSK9 tertiles

**Fig. 2:**
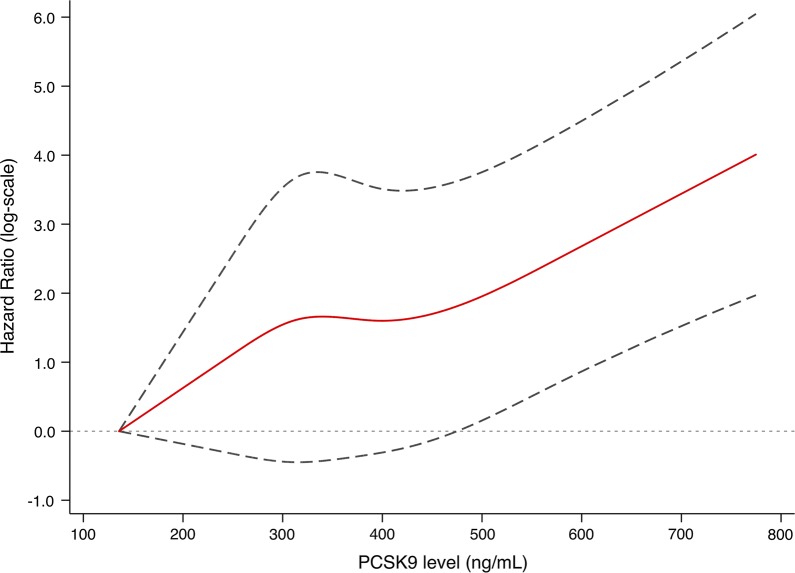
Adjusted restricted cubic spline plot of the hazard ratio of MACE against PCSK9 levels

Kaplan–Meier curve showing the probability of CVD event-free survival during the follow-up period according to PCSK9 tertiles is presented in Fig. 1. Patients with higher levels of PCSK9 had lower event-free survival rates (p = 0.0017). Univariate Cox regression analyses revealed that PCSK9 was associated with MACEs when upper vs lower tertile was compared (HR: 4.44, 95 CI% 1.62–12.18; Table 3). After adjustment for age and sex, higher plasma PCSK9 levels were significantly associated with a higher rate of events. In the fully adjusted model, patients with PCSK9 concentrations in tertile 3 had more than a 3.7-fold higher risk of MACEs than patients in tertile 1 (HR: 3.70, 95 CI% 1.16–11.82). Likewise, on a continuous scale, each SD increase of PCSK9 concentration was significantly associated with an 86% risk increase for MACEs (HR: 1.86, 95 CI% 1.31–2.65). The results of the spline analysis are presented in Fig. 2. There was an increasing nonlinear trend in PCSK9 with MACEs. In sensitivity analyses, the results were persisted when forced each of the other variables into the model with PCSK9 (Additional file 1: Table S8). The Cox prediction model consisting traditional risk factors was with a C-statistic of 0.652 (95% CI 0.549–0.754), whereas adding PCSK9 to the risk prediction model increased the C-statistic to 0.682 (95% CI 0.583–0.780; Additional file 1: Fig. S4). Furthermore, adding PCSK9 to model also resulted in a significant increase in continuous NRI (34.1%, 95 CI% 3.2%–56.8%, p = 0.041) and IDI (10.8%, 95 CI% 1.0%–35.7%, p = 0.010).

**Table 3:** Multivariate Cox proportional hazards regression analysis of MACEs

Model	PCSK9 1-SD increase	PCSK9 T1	PCSK9 T2	PCSK9 T3
Crude	1.92 (1.43–2.57)^†^	1.00 (Reference)	2.57 (0.90–7.33)	4.44 (1.62–12.18)^†^
Model 1	1.99 (1.46–2.68)^†^	1.00 (Reference)	2.76 (0.96–7.95)	4.85 (1.74–13.54)^†^
Model 2	1.95 (1.41–2.70)^†^	1.00 (Reference)	2.94 (1.00–8.63)	3.93 (1.35–11.42)^*^
Model 3	1.86 (1.29–2.69)^*^	1.00 (Reference)	3.36 (1.11–10.22)^*^	3.76 (1.16–12.27)^*^
Model 4	1.86 (1.31–2.65)^*^	1.00 (Reference)	3.30 (1.09–10.01)^*^	3.70 (1.16–11.82)^*^

^*^p < 0.05; ^†^p < 0.01

Model 1 adjusted for age and sex; Model 2 adjusted for Model 1 plus body mass index, baseline statin use, prior coronary artery disease, hypertension, diabetes and current smoking; Model 3 adjusted for Model 2 plus low- and high-density lipoprotein cholesterol, total cholesterol, apolipoprotein B, lipoprotein (a) and high sensitivity C-reactive protein; Model 4 adjusted for Model 3 plus genetic mutations. PCSK9, proprotein convertase subtilisin-kexin type 9; MACE: major adverse cardiovascular events

**Fig. 3:**
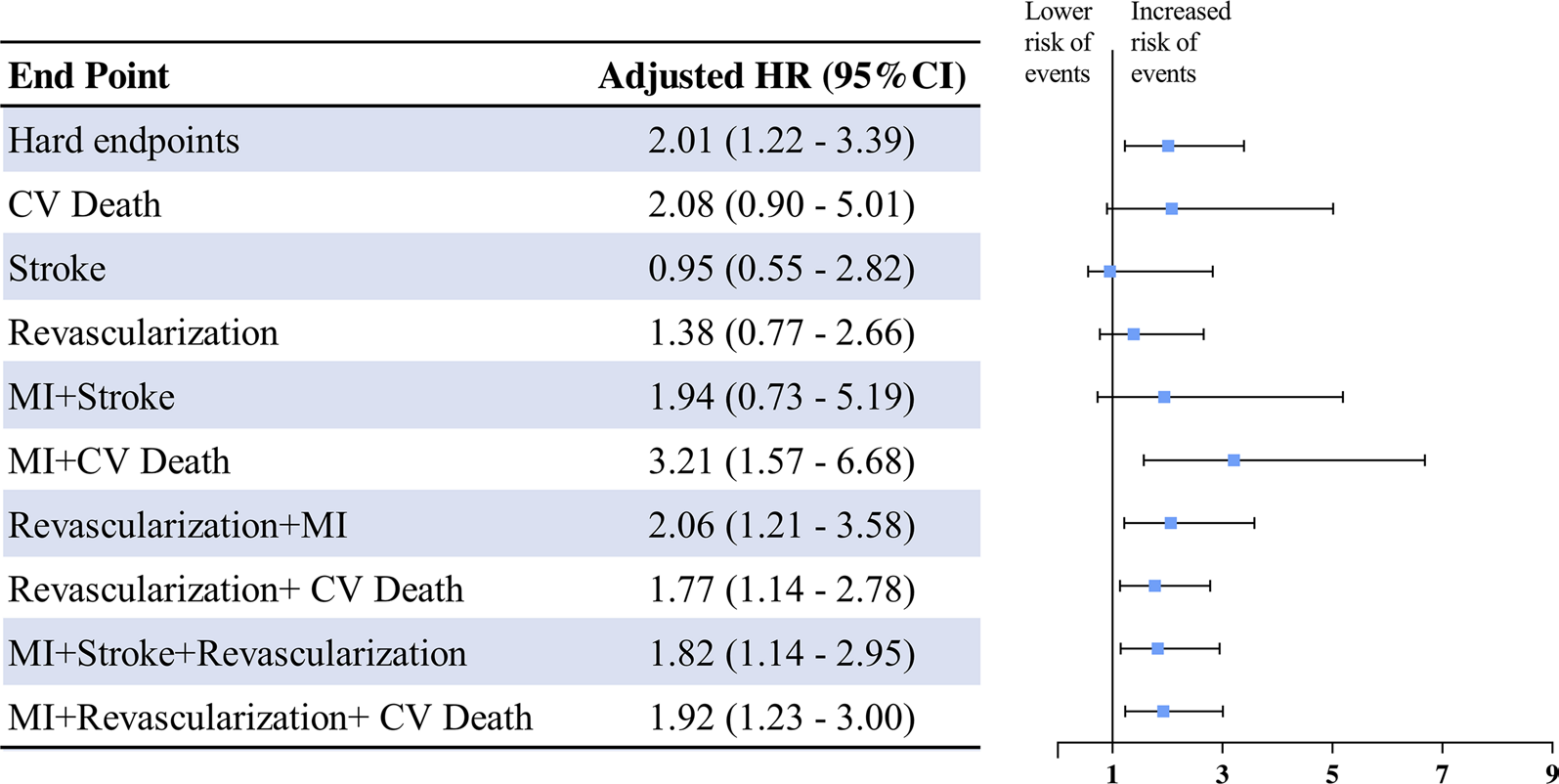
Hazards ratios for cardiovascular events per SD higher PCSK9 level, adjusted for cardiovascular risk factors

For hard endpoints, a more pronounced HR was found between PCSK9 levels and events (HR: 2.01, 95 CI% 1.22–3.39), as shown in Fig. 3. Findings were broadly similar when the outcomes of MI, stroke, revascularization and cardiovascular death were combined. The association between PCSK9 and stroke showed an adverse but not statistically significant difference (HR: 0.95, 95 CI% 0.55–2.82). Subgroup analyses were then performed according to some clinical characteristics. As shown in Additional file 1: Table S9, a positive association between serum PCSK9 levels and incident MACEs was observed in all subgroups. However, in patients with DM, the association was not statistically significant (p for interaction = 0.031).

## Discussion

In this prospective cohort of patients with FH, we investigated the association of plasma PCSK9 levels with the calcification and stenosis in coronary atherosclerosis and future cardiovascular events under the condition of the era of optimal lipid-lowering therapy. The main finding was that higher serum PCSK9 levels were associated with a higher CACS and Gensini Score. Besides, plasma PCSK9 concentration at baseline was associated with risk of incident MACEs even after adjustment for established cardiovascular risk factors. Adding PCSK9 to risk prediction model could provide incremental information for MACEs. To the best of our knowledge, this is the first study to indicate that circulating PCSK9 concentration was a useful marker for predicting outcome in patients with FH, even in ones receiving standard lipid-lowering therapy, suggesting the measurement of PCSK9 might help to further stratify the risk of FH patients clinically.

PCSK9 is well-known for its role in the regulation of cholesterol homeostasis currently [1, 3]. Moreover, recent experimental and clinical studies have also reported that higher circulating PCSK9 level contributed to coronary atherosclerosis via enhancing the expression of pro-inflammatory genes, promoting apoptosis of human endothelial cells and activating platelet reactivity [7, 27, 28]. More importantly, some cross-sectional studies have also shown that high circulating PCSK9 levels accelerated the development of coronary, carotid and femoral atherosclerosis [10, 22, 24]. For example, Alonso et al. reported that PCSK9 was independently associated with higher CACS in asymptomatic FH patients [29]. The present study was in line with previous studies that PCSK9 was associated with a higher risk of coronary calcification and stenosis in patients with FH after adjusting for other traditional cardiovascular risk factors, suggesting that circulating PCSK9 might be a useful biomarker concerning coronary atherosclerosis.

In view of the functional diversity of PCSK9, its circulating concentration was considered as a potential atherogenic risk marker for cardiovascular events [28, 30]. For example, Leander et al. [9] enrolled 4232 general population and found a positive correlation between PCSK9 plasma concentration and incident MACEs. Our previous prospective observational study also suggested that circulating PCSK9 levels could predict future of cardiovascular events in patients with stable CAD [10]. Although the relationship between PCSK9 concentration and incident cardiac events in general population has been studied, this association in FH patients has yet to be evaluated.

FH is a special group of population who has genetic mutations resulting in lifetime elevated plasma LDL-C levels and accelerated atherosclerosis. Their clinical characteristics were quite heterogeneous, making it difficult to stratify the patients for primary and secondary prevention and treatment [31]. Indeed, it was reported that the predictive value of some traditional risk factor for future MACEs were different from the general population [31]. For instance, in the Montreal-FH-SCORE study the investigators found that HDL-C was a useful predictor for clinical outcomes but not LDL-C [18]. Likewise, a recent study showed that baseline clinical characteristic and lipid profile in FH patients were on longer associated with incident MACEs in multivariate Cox regression analysis [32]. Hence, the exploration of prognostic predicator for FH patients may be of great interest. Notably, PCSK9 level has been consistently reported to be significantly higher in patients with FH comparing with normocholesterolemic participants and higher circulating PCSK9 level might contribute to a worse hypercholesterolemic phenotype in FH patients independent of *LDLR* genotype [33, 34]. For example, Drouin-Chartier et al. has reported that mean PCSK9 levels were significantly higher in the HeFH group than in the control group (317.9 vs. 203.3 ng/mL) [33]. In view of its multiple function, higher level in FH and contribution to FH phenotype as well as no relevant research, we hypothesized that circulating PCSK9 might act as a novel predictor and therefore examined the impact of PCSK9 on the cardiovascular outcomes of patients with FH in this prospective cohort.

In the present study, we found that elevated PCSK9 was associated with a worse prognosis in patients with FH and the association was persisted after adjustment for multiple established CAD risk factors and baseline statin use. Interestingly, when the outcome of stroke was evaluated, the association showed an adverse and non-significant result. In fact, previous studies have provided divergent results regarding whether *PCSK9* gene is associated with high risk of stroke and only one study evaluated the relationship between circulating PCSK9 and stroke, and found non-significant association in large cohorts [35, 36]. Besides, it was reported that statins activate the expression of *PCSK9* genes and increase plasma PCSK9 levels [37, 38]. In the present study, all the FH patients received standard statin therapy after discharged and we found that baseline PCSK9 levels could predict future MACEs and the association was persisted in subgroup analyses stratified by baseline statin use. Moreover, we observed the ascending trend of PCSK9 levels in drinkers. An experimental study has showed that chronic alcohol feeding increased gene expression of PCSK9 in rats [39]. However, no relevant data was available in human currently. Consequently, our study might provide novel information regarding the prognostic role of PCSK9 in patients with FH receiving standard lipid-lowering therapy. Nevertheless, the future analyses are needed to allow us to gain further understanding of the role of PCSK9 in cardiovascular outcomes in patients with FH.

In this study, we assessed the incremental value of the addition of PCSK9 levels in a risk prediction model with established risk factors using C-statistics, continuous NRI and IDI. The results of these analyses suggested that plasma PCSK9 levels could provide incremental information for MACEs. Moreover, the identification of increased individual PCSK9 level might have clinical utility in further risk stratification of MACEs in patients with FH and provide guidance to therapies. Considering the high price of PCSK9 inhibitors, PCSK9 assessment may help further identify the patients who may derive greater benefits from PCSK9 inhibitors. Unquestionably, more studies about the association between PCSK9 and MACEs may be needed considering our relatively small sample size.

Our study has several limitations. First, the number of overall populations in the study was relatively small and the statistical power might be insufficient in some subgroup analyses. However, our results were significant and robust. Second, circulating PCSK9 was measured at a single point and the longitudinal trajectory of change in PCSK9 during the follow-up might provide additional information. Additionally, attributing to the lack of a ‘gold standard’ for test circulating PCSK9, the ELISA assay used in our study was for total circulating PCSK9 like most studies. We could not exclude the theoretical possibility that different PCSK9 forms might have different correlations with future MACEs. Moreover, although we enrolled clinical diagnosed FH patients (probably/definite), a part of them did not achieved “definite” according to DLCN. Finally, although multivariable Cox regression analyses including several confounding variables were performed, the possibility of residual confounding cannot be precluded. It is noticeable that, the current study on PCSK9 was an exploratory research and further steps are still needed to confirm its prognostic value in FH patients.

## Conclusions

In conclusion, the present study indicates that higher levels of PCSK9 at baseline were significantly correlated with coronary atherosclerosis. More importantly, high PCSK9 was found to be positively associated with future adverse cardiovascular outcomes in definite/probable FH patients with standard lipid-lowering therapy, indicating that assessing PCSK9 levels in patients with FH may be useful for cardiovascular risk stratification.

## Supplementary information

**Additional file 1: Table S1.** Dutch Lipid Clinic Network Clinical Criteria for familial hypercholesterolemia. **Table S2.** International Classification of Diseases Codes for MACE. **Table S3.** Correlation of PCSK9 level with clinical characteristics at baseline. **Table S4.** Baseline characteristics of FH patients according to CACS. **Table S5.** Baseline characteristics of FH patients according to Gensini Score. **Table S6.** Correlations between CACS, Gensini Score and variables. **Table S7.** Multiple linear regression analyses of CACS, Gensini score and PCSK9. **Table S8.** Sensitivity analysis of the association of PCSK9 with primary composite endpoints after separate adjustment for each of the other significantly variables. **Table S9.** Adjusted hazard ratio and 95% confidence intervals of cardiovascular events according to plasma PCSK9 per-SD increase: subgroup analyses. **Figure S1.** Comparison of PCSK9 concentrations between patients with or without an event. Values are presented as median ± SD. **Figure S2.** Correlation of plasma PCSK9 levels with other biomarkers. **Figure S3.** Relationship between CACS, Gensini score and PCSK9 concentration. **Figure S4.** Receiver operating characteristic curve.

## Data Availability

All data generated or analysed during this study are included in this published article and its supplementary information files.

## Abbreviations

PCSK9: proprotein convertase subtilisin/kexin 9
CAD: coronary artery disease
FH: familial hypercholesterolemia
MACE: major cardiovascular event
CACS: coronary artery calcification score
GS: Gensini score
HR: hazard ratio
CI: confidence interval
LDL-C: low-density lipoprotein cholesterol

## References

[1] Burke AC, Dron JS, Hegele RA, Huff MW (2017). PCSK9: regulation and target for drug development for dyslipidemia. Annu Rev Pharmacol Toxicol.

[2] Spolitu S, Okamoto H, Dai W (2019). Hepatic glucagon signaling regulates PCSK9 and low-density lipoprotein cholesterol. Circ Res.

[3] Gustafsen C, Olsen D, Vilstrup J (2017). Heparan sulfate proteoglycans present PCSK9 to the LDL receptor. Nat Commun.

[4] Bonaca MP, Nault P, Giugliano RP (2018). Low-density lipoprotein cholesterol lowering with evolocumab and Outcomes in patients With peripheral artery disease: insights from the FOURIER Trial (further cardiovascular outcomes research with PCSK9 inhibition in subjects with elevated risk). Circulation.

[5] Szarek M, White HD, Schwartz GG (2019). Alirocumab reduces total nonfatal cardiovascular and fatal events: The ODYSSEY OUTCOMES trial. J Am Coll Cardiol.

[6] Cao YX, Liu HH, Li S, Li JJ (2019). A meta-analysis of the effect of PCSK9-monoclonal antibodies on circulating lipoprotein (a) levels. Am J Cardiovasc Drugs.

[7] Ferri N, Marchiano S, Tibolla G (2016). PCSK9 knock-out mice are protected from neointimal formation in response to perivascular carotid collar placement. Atherosclerosis.

[8] Tang Z, Jiang L, Peng J (2012). PCSK9 siRNA suppresses the inflammatory response induced by oxLDL through inhibition of NF-kappaB activation in THP-1-derived macrophages. Int J Mol Med.

[9] Leander K, Malarstig A, Van’t Hooft FM (2016). Circulating proprotein convertase subtilisin/kexin type 9 (PCSK9) predicts future risk of cardiovascular events independently of established risk factors. Circulation.

[10] Li JJ, Li S, Zhang Y (2015). Proprotein convertase subtilisin/kexin type 9, C-reactive protein, coronary severity, and outcomes in patients with stable coronary artery disease: a prospective observational cohort study. Medicine.

[11] Ridker PM, Rifai N, Bradwin G, Rose L (2016). Plasma proprotein convertase subtilisin/kexin type 9 levels and the risk of first cardiovascular events. Eur Heart J.

[12] Zhu YM, Anderson TJ, Sikdar K (2015). Association of proprotein convertase subtilisin/kexin type 9 (PCSK9) with cardiovascular risk in primary prevention. Arterioscler Thromb Vasc Biol.

[13] Sturm AC, Knowles JW, Gidding SS (2018). Clinical genetic testing for familial hypercholesterolemia: JACC Scientific Expert Panel. J Am Coll Cardiol.

[14] Li JJ, Li S, Zhu CG (2017). Familial hypercholesterolemia phenotype in chinese patients undergoing coronary angiography. Arterioscler Thromb Vasc Biol.

[15] Nordestgaard BG, Chapman MJ, Humphries SE (2013). Familial hypercholesterolaemia is underdiagnosed and undertreated in the general population: guidance for clinicians to prevent coronary heart disease: consensus statement of the European Atherosclerosis Society. Eur Heart J.

[16] Tscharre M, Herman R, Rohla M (2019). Prognostic impact of familial hypercholesterolemia on long-term outcomes in patients undergoing percutaneous coronary intervention. J Clin Lipidol.

[17] Cao YX, Wu NQ, Sun D (2018). Application of expanded genetic analysis in the diagnosis of familial hypercholesterolemia in patients with very early-onset coronary artery disease. J Transl Med.

[18] Paquette M, Dufour R, Baass A (2017). The Montreal-FH-SCORE: a new score to predict cardiovascular events in familial hypercholesterolemia. J Clin Lipidol.

[19] Lambert G, Petrides F, Chatelais M (2014). Elevated plasma PCSK9 level is equally detrimental for patients with nonfamilial hypercholesterolemia and heterozygous familial hypercholesterolemia, irrespective of low-density lipoprotein receptor defects. J Am Coll Cardiol.

[20] Besseling J, Kindt I, Hof M, Kastelein JJ, Hutten BA, Hovingh GK (2014). Severe heterozygous familial hypercholesterolemia and risk for cardiovascular disease: a study of a cohort of 14,000 mutation carriers. Atherosclerosis.

[21] Sun D, Li S, Zhao X (2018). Association between lipoprotein (a) and proprotein convertase substilisin/kexin type 9 in patients with heterozygous familial hypercholesterolemia: a case-control study. Metabolism.

[22] Cao YX, Liu HH, Sun D (2018). The different relations of PCSK9 and Lp(a) to the presence and severity of atherosclerotic lesions in patients with familial hypercholesterolemia. Atherosclerosis.

[23] Richards S, Aziz N, Bale S (2015). Standards and guidelines for the interpretation of sequence variants: a joint consensus recommendation of the American College of Medical Genetics and Genomics and the Association for Molecular Pathology. Genet Med.

[24] Zhao X, Zhang HW, Li S (2018). Association between plasma proprotein convertase subtisilin/kexin type 9 concentration and coronary artery calcification. Ann Clin Biochem.

[25] Agatston AS, Janowitz WR, Hildner FJ, Zusmer NR, Viamonte M, Detrano R (1990). Quantification of coronary artery calcium using ultrafast computed tomography. J Am Coll Cardiol.

[26] Li S, Zhang Y, Xu RX (2015). Proprotein convertase subtilisin-kexin type 9 as a biomarker for the severity of coronary artery disease. Ann Med.

[27] Ferri N, Tibolla G, Pirillo A (2012). Proprotein convertase subtilisin kexin type 9 (PCSK9) secreted by cultured smooth muscle cells reduces macrophages LDLR levels. Atherosclerosis.

[28] Navarese EP, Kolodziejczak M, Winter MP (2017). Association of PCSK9 with platelet reactivity in patients with acute coronary syndrome treated with prasugrel or ticagrelor: the PCSK9-REACT study. Int J Cardiol.

[29] Alonso R, Mata P, Muñiz O (2016). PCSK9 and lipoprotein (a) levels are two predictors of coronary artery calcification in asymptomatic patients with familial hypercholesterolemia. Atherosclerosis.

[30] Werner C, Hoffmann MM, Winkler K, Bohm M, Laufs U (2014). Risk prediction with proprotein convertase subtilisin/kexin type 9 (PCSK9) in patients with stable coronary disease on statin treatment. Vascul Pharmacol.

[31] Sharifi M, Rakhit RD, Humphries SE, Nair D (2016). Cardiovascular risk stratification in familial hypercholesterolaemia. Heart.

[32] Miname MH, Bittencourt MS, Moraes SR (2019). Coronary artery calcium and cardiovascular events in patients with familial hypercholesterolemia receiving standard lipid-lowering therapy. JACC Cardiovasc Imaging.

[33] Cameron J, Bogsrud MP, Tveten K (2012). Serum levels of proprotein convertase subtilisin/kexin type 9 in subjects with familial hypercholesterolemia indicate that proprotein convertase subtilisin/kexin type 9 is cleared from plasma by low-density lipoprotein receptor-independent pathways. Transl Res.

[34] Drouin-Chartier JP, Tremblay AJ, Hogue J-C, Ooi TC, Lamarche B, Couture P (2015). The contribution of PCSK9 levels to the phenotypic severity of familial hypercholesterolemia is independent of LDL receptor genotype. Metabolism.

[35] Slimani A, Harira Y, Trabelsi I (2014). Effect of E670G Polymorphism in PCSK9 gene on the risk and severity of coronary heart disease and ischemic stroke in a Tunisian Cohort. J Mol Neurosci.

[36] El Khoury P, Roussel R, Fumeron F (2018). Plasma proprotein convertase subtilisin/kexin type 9 (PCSK9) and cardiovascular events in type 2 diabetes. Diabetes Obes Metab.

[37] Awan Z, Seidah NG, MacFadyen JG (2012). Rosuvastatin, proprotein convertase subtilisin/kexin type 9 concentrations, and LDL cholesterol response: the JUPITER trial. Clin Chem.

[38] Guo YL, Liu J, Xu RX (2013). Short-term impact of low-dose atorvastatin on serum proprotein convertase subtilisin/kexin type 9. Clin Drug Investig.

[39] Wang Z, Yao T, Song Z (2010). Chronic alcohol consumption disrupted cholesterol homeostasis in rats: down-regulation of low-density lipoprotein receptor and enhancement of cholesterol biosynthesis pathway in the liver. Alcohol Clin Exp Res.

