# Supplementary material for: Circulating PCSK9 and cardiovascular events in FH patients with standard lipid-lowering therapy
Source: J Transl Med. 2019 Nov 11;17:367. doi: 10.1186/s12967-019-2123-9 (PMC6849213; doi:10.1186/s12967-019-2123-9)
Supplement: Supplementary file 1 — Additional file 1: Table S1. Dutch Lipid Clinic Network Clinical Criteria for familial hypercholesterolemia. Table S2. International Classification of Diseases Codes for MACE. Table S3. Correlation of PCSK9 level with clinical characteristics at baseline. Table S4. Baseline characteristics of FH patients according to CACS. Table S5. Baseline characteristics of FH patients according to Gensini Score. Table S6. Correlations between CACS, Gensini Score and variables. Table S7. Multiple linear regression analyses of CACS, Gensini score and PCSK9. Table S8. Sensitivity analysis of the association of PCSK9 with primary composite endpoints after separate adjustment for each of the other significantly variables. Table S9. Adjusted hazard ratio and 95% confidence intervals of cardiovascular events according to plasma PCSK9 per-SD increase: subgroup analyses. Figure S1. Comparison of PCSK9 concentrations between patients with or without an event. Values are presented as median ± SD. Figure S2. Correlation of plasma PCSK9 levels with other biomarkers. Figure S3. Relationship between CACS, Gensini score and PCSK9 concentration. Figure S4. Receiver operating characteristic curve. [file 12967_2019_2123_MOESM1_ESM.pdf]

SUPPLEMENTAL MATERIAL

Table S1. Dutch Lipid Clinic Network Clinical Criteria for familial hypercholesterolemia

Criteria	Points
Family history	
First-degree relative with known premature (men: <55 years; women: <60 years) coronary artery disease or vascular disease	1
First-degree relative with known LDL-C level above the 95th percentile	1
First-degree relative with tendinous xanthomata and/or arcus cornealis	2
Children aged less than 18 years with LDL-C level above the 95th percentile	2
Clinical history	
Patient with premature (men: <55 years; women: <60 years) coronary artery disease	2
Patient with premature (men: <55 years; women: <60 years) cerebrovascular or peripheral vascular disease	1
Physical examination	
Tendinous xanthomata	6
Arcus cornealis (<45 years)	4
LDL-C levels	
LDL-C ≥8.5 mmol/l	8
LDL-C 6.5–8.4 mmol/l	5
LDL-C 5.0–6.4 mmol/l	3
LDL-C 4.0–4.9 mmol/l	1
DNA analysis	
Causative mutation in the *LDLR, ApoB* or *PCSK9* gene	8

Total score of diagnosis FH:

Definite FH >8

Probable FH 6–8

Possible FH 3–5

Unlikely FH 0–2

Table S2. International Classification of Diseases Codes for MACE.

**Diagnosis/Procedure**	**ICD-9-CM codes/ ICD-10 codes**
myocardial infarction	I21
angina pectoris	I20
cardiovascular death	death due to I00-I10, I11,I13, I20-I51
ischemic stroke	I63, I64
percutaneous coronary intervention	ICD-9-CM 36.06/36.07
coronary artery bypass grafting	ICD-9-CM 36.1-36.19

Table S3. Correlation of PCSK9 level with clinical characteristics at baseline.

**variables**	**Spearman rho**	**p value**
Age	0.009	0.873
Male	0.153	0.005
BMI	-0.056	0.340
Family history of CHD	0.014	0.794
Currently smoking	0.038	0.517
Alcohol drinker	0.132	0.122
Hypertension	0.031	0.587
Diabetes	0.057	0.311
Baseline statin use	0.036	0.504
XM	0.21	0.001
TG	-0.061	0.263
TC	0.235	0.001
HDL-C	0.012	0.828
LDL-C	0.227	0.001
ApoA	-0.026	0.642
ApoB	0.221	0.001
Lp(a)	0.256	0.001
hsCRP	0.002	0.977
FPG	0.007	0.897
HbA1C	0.018	0.761
WBC	0.015	0.797
LYM	-0.023	0.700

Table S4. Baseline characteristics of FH patients according to CACS.

Variables	CACS 0 (N=28)	CACS 1-100 (N=71)	CACS >100 (N=63)	p-value for trend
Clinical factors				
Age, years	52.31 ± 8.14	50.04 ± 10.39	51.22 ± 10.29	0.541
Male, n (%)	17 (60.7)	45 (63.4)	32 (50.8)	0.473
BMI, kg/(m^2)	25.31 ± 2.58	25.64 ± 3.67	25.98 ± 3.30	0.652
Family history of CAD, n (%)	16 (57.1)	36 (50.7)	31 (49.2)	0.551
Currently smoking, n (%)	12 (42.9)	35 (49.3)	28 (44.4)	0.345
Alcohol drinker, n (%)	8 (28.6)	21 (29.6)	16 (25.4)	0.882
Hypertension, n (%)	13 (40.6)	35 (49.3)	36 (61.0)	0.151
Diabetes, n (%)	4 (14.3)	10 (14.1)	12 (19.5)	0.520
Baseline statin use, n (%)	24 (85.7)	64 (90.1)	49 (77.8)	0.402
Tendon xanthoma, n (%)	2 (7.1)	5 (7.0)	3 (4.8)	0.899
Laboratory factors				
TG, mmol/L	1.71 (1.30 - 2.08)	1.58 (1.24 - 1.85)	1.71 (1.26 - 2.36)	0.058
TC, mmol/L	6.04 ± 1.52	6.11 ± 1.43	7.12 ± 1.97	0.002
HDL-C, mmol/L	1.12 ± 0.27	1.08 ± 0.34	1.08 ± 0.29	0.802
LDL-C, mmol/L	4.41 ± 1.59	4.51 ± 1.25	5.31 ± 1.68	0.003
ApoA, g/L	1.34 ± 0.27	1.30 ± 0.32	1.31 ± 0.33	0.774
ApoB, g/L	1.34 ± 0.38	1.31 ± 0.36	1.54 ± 0.49	0.006
Lp(a), mg/dL	27.76 (13.69-50.97)	43.88 (19.40-67.41)	37.94 (11.04-82.27)	0.303
hsCRP, mg/L	2.11 (0.99 - 3.56)	1.58 (0.79 - 3.68)	1.58 (0.75 - 3.54)	0.941
FPG, mmol/L	5.86 ± 2.09	5.30 ± 1.55	5.75 ± 1.42	0.155
HbA1C, %	6.24 ± 1.35	6.14 ± 1.09	6.28 ± 1.10	0.782
WBC, ng/mL	6.54 ± 2.01	6.41 ± 1.90	6.69 ± 1.98	0.713
LYM, ng/mL	1.95 ± 0.65	2.02 ± 0.51	2.11 ± 0.70	0.442
PCSK9, ng/mL	240.71 (216.74 - 309.49)	291.58 (239.09 - 367.61)	377.68 (318.51 - 447.64)	< 0.001

Table S5. Baseline characteristics of FH patients according to Gensini Score.

Variables	GS T1 (N=94)	GS T2 (N=95)	GS T3 (N=95)	p-value for trend
Clinical factors				
Age, years	49.57 ± 11.04	51.98 ± 9.27	49.23 ± 10.78	0.141
Male, n (%)	50 (53.2)	57 (60.0)	62 (65.3)	0.238
BMI, kg/(m^2)	24.86 ± 3.12	25.81 ± 3.24	25.98 ± 3.62	0.066
Family history of CAD, n (%)	38 (40.4)	48 (50.5)	47 (49.5)	0.311
Currently smoking, n (%)	24 (25.5)	49 (51.6)	42 (44.2)	0.022
Alcohol drinker, n (%)	14 (14.9)	33 (34.7)	23 (24.2)	0.034
Hypertension, n (%)	23 (24.5)	51 (53.7)	54 (56.8)	0.001
Diabetes, n (%)	14 (14.9)	17 (17.9)	25 (26.3)	0.123
Statin use, n (%)	60 (63.8)	91 (95.8)	84 (88.4)	0.001
Tendon xanthoma, n (%)	7 (7.4)	4 (4.2)	11 (11.6)	0.163
Laboratory factors				
TG, mmol/L	1.51 (1.11 - 2.10)	1.71 (1.30 - 2.14)	1.72 (1.28 - 2.35)	0.303
TC, mmol/L	7.24 ± 2.04	6.15 ± 1.42	7.06 ± 2.22	0.000
HDL-C, mmol/L	1.19 ± 0.36	1.11 ± 0.30	1.04 ± 0.28	0.005
LDL-C, mmol/L	5.25 ± 1.78	4.53 ± 1.31	5.22 ± 1.75	0.003
ApoA, g/L	1.38 ± 0.30	1.33 ± 0.31	1.27 ± 0.31	0.077
ApoB, g/L	1.47 ± 0.45	1.33 ± 0.33	1.50 ± 0.49	0.015
Lp(a), mg/dL	25.95 (10.6 - 57.62)	35.74 (12.06 - 65.94)	40.83 (18.93 - 81.67)	0.036
HsCRP, mg/L	2.23 ± 2.73	2.71 ± 2.79	2.90 ± 3.17	0.261
FPG, mmol/L	5.18 ± 0.84	5.67 ± 1.64	5.91 ± 1.89	0.362
HbA1C, %	5.83 ± 0.66	6.19 ± 1.05	6.40 ± 1.30	0.003
WBC, ng/mL	6.14 ± 1.56	6.40 ± 1.86	6.51 ± 1.98	0.413
LYM, ng/mL	2.08 ± 0.69	2.03 ± 0.56	2.03 ± 0.77	0.870
PCSK9, ng/mL	299.02 (218.16 - 355.18)	306.38 (241.62 - 387.57)	331.50 (260.41 - 430.49)	0.025

Table S6. Correlations between CACS, Gensini Score and variables.

Variables	CACS		Gensini Score	
	Spearman rho	p-value	Spearman rho	p-value
Age	0.050	0.527	0.006	0.919
Male	0.053	0.501	0.07	0.237
BMI	0.061	0.443	0.104	0.089
Family history of CAD	0.088	0.263	0.07	0.237
Currently smoking	0.079	0.316	0.066	0.285
Alcohol drinker	0.018	0.821	0.045	0.464
Hypertension	0.148	0.060	0.274	0.001
Diabetes	0.110	0.162	0.138	0.020
Baseline statin use	0.096	0.224	-0.277	0.001
Tendon xanthoma	-0.017	0.829	0.069	0.248
TG	0.079	0.319	0.099	0.095
TC	0.357	0.001	-0.057	0.340
HDL-C	-0.001	0.989	-0.176	0.003
LDL-C	0.346	0.001	-0.037	0.531
ApoA	-0.046	0.563	-0.12	0.044
ApoB	0.265	0.001	0.001	0.987
Lp(a)	0.018	0.824	0.171	0.004
hsCRP	-0.010	0.899	0.161	0.007
FPG	0.103	0.193	0.145	0.014
HbA1C	0.119	0.135	0.178	0.004
WBC	0.073	0.360	0.068	0.272
LYM	0.073	0.356	-0.077	0.215
PCSK9	0.504	0.001	0.426	0.022

Table S7. Multiple linear regression analyses of CACS, Gensini score and PCSK9.

**Variables**	**CACS**				**Gensini score**			
	**Univariate**		**Multivariate**		**Univariate**		**Multivariate**	
	**β**	**p-value**	**β**	**p-value**	**β**	**p-value**	**β**	**p-value**
Age	-0.015	0.985			-6.224	0.290		
Male	3.710	0.819			-0.154	0.580		
BMI	2.066	0.393			1.397	0.120		
Family history of CAD	12.74	0.425			8.726	0.132		
Currently smoking	10.057	0.530			1.604	0.860		
Hypertension	28.763	0.070			21.783	0.001	19.714	0.001
Diabetes	31.027	0.152	39.565	0.034	19.342	0.010		
Baseline statin use	-25.306	0.296			-22.083	0.001	-17.208	0.05
TG	11.450	0.077			0.358	0.709		
TC	19.595	0.000	10.161	0.018	1.749	0.230		
HDL-C	1.500	0.954			-26.954	0.001		
LDL-C	21.606	0.000			2.140	0.220	5.623	0.003
ApoB	65.916	0.000			12.960	0.050		
Lp(a)	-0.037	0.877			0.196	0.030	0.196	0.020
hsCRP	1.893	0.472			3.023	0.000		
FPG	3.896	0.425			5.853	0.001		
HbA1C	2.747	0.691			8.641	0.001	7.242	0.005
PCSK9	0.483	0.000	0.449	< 0.001	0.422	0.001	0.435	0.014

Table S8. Sensitivity analysis of the association of PCSK9 with primary composite endpoints after separate adjustment for each of the other significantly variables.

**Adjustment variable**	**Multivariable analysis**	
	**Hazard ratio for PCSK9-SD increase**	**p-value**
LDL-C	2.00 (1.46 - 2.73)	<0.001
Lp(a)	1.82 (1.35 - 2.46)	<0.001

Table S9. Adjusted hazard ratio and 95% confidence intervals of cardiovascular events according to plasma PCSK9 per-SD increase: Subgroup Analyses

**Characteristics**	**Case**	**No. of participants**	**Adjusted HR (95%CI)**	**p-value for per SD**	**p-value for interaction**
Sex					0.802
Female	13	140	2.85 (1.18-6.42)	0.014	
Male	20	198	2.25 (1.43-3.53)	0.001	
Age					0.824
<55	22	231	1.93 (1.26-3.06)	0.008	
≥ 55	11	107	5.38 (1.63-17.73)	0.011	
Baseline Statin use					0.292
yes	28	262	2.06 (1.31-3.22)	0.005	
no	5	76	2.25 (1.56-3.24)	0.019	
BMI					0.156
< 24	26	209	3.72 (1.14-7.34)	0.030	
≥ 24	7	129	1.67 (1.12-2.50)	0.021	
Diabetes					0.031
yes	6	64	1.61 (0.58-5.83)	0.588	
No	27	274	2.35 (1.61-3.43)	0.001	
Hypertension					0.188
yes	21	140	1.71 (1.02-2.92)	0.044	
No	12	198	3.35 (1.69-6.67)	0.001	
Family history of CAD					0.222
yes	12	157	2.32 (1.04-5.20)	0.045	
No	21	181	2.78 (1.80-4.29)	0.001	
LDL-C					0.620
<4.9	18	179	1.81 (1.02-3.37)	0.043	
≥ 4.9	15	168	2.13 (1.20-3.78)	0.010	

Figure S1. Comparison of PCSK9 concentrations between patients with or without an event. Values are presented as median ± SD.

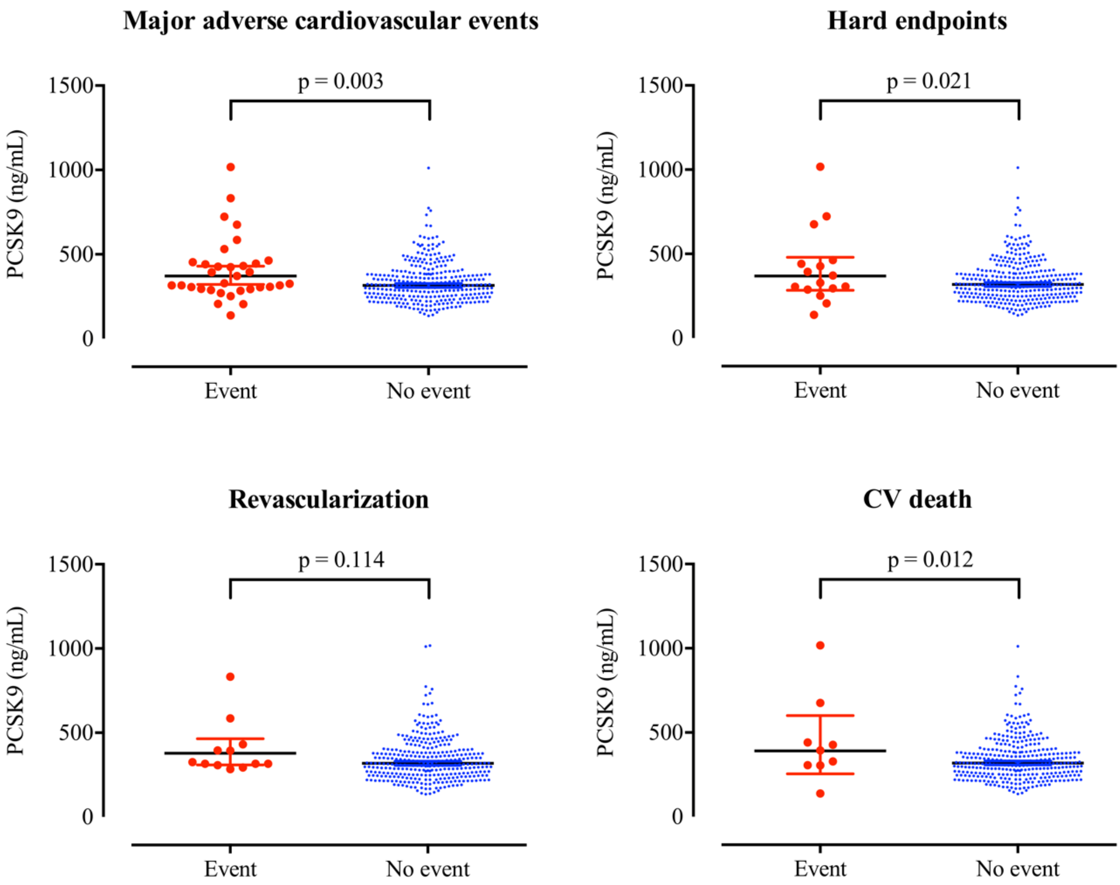

Figure S2. Correlation of plasma PCSK9 levels with other bio-markers.

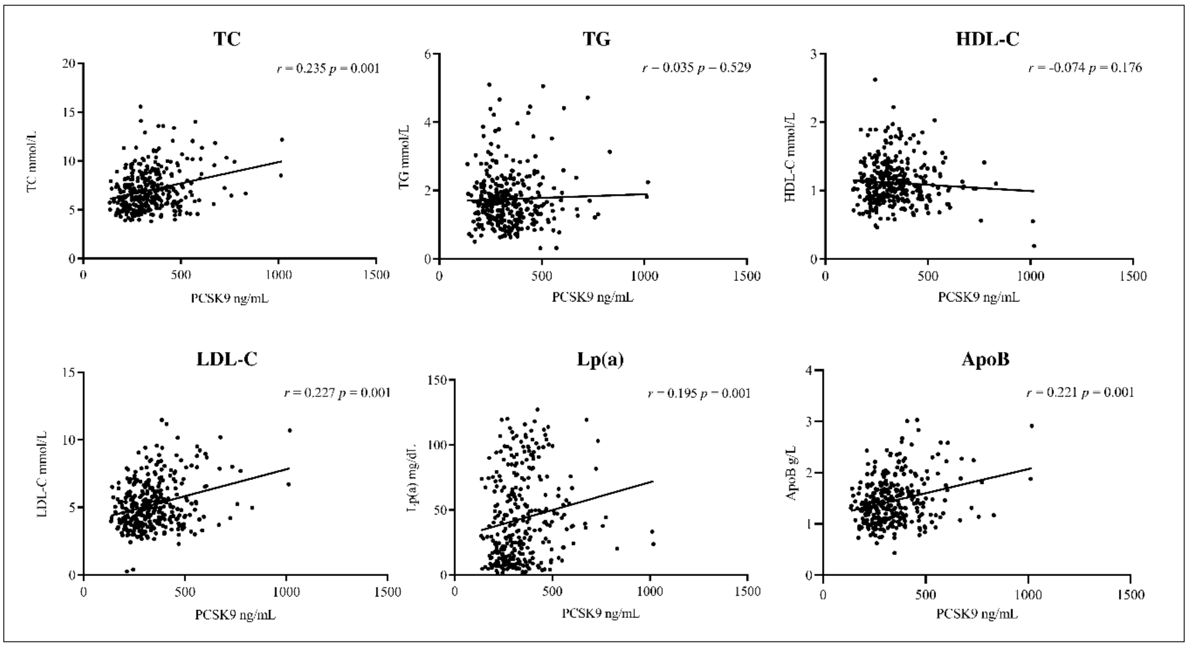

Figure S3. Relationship between CACS, Gensini score and PCSK9 concentration.

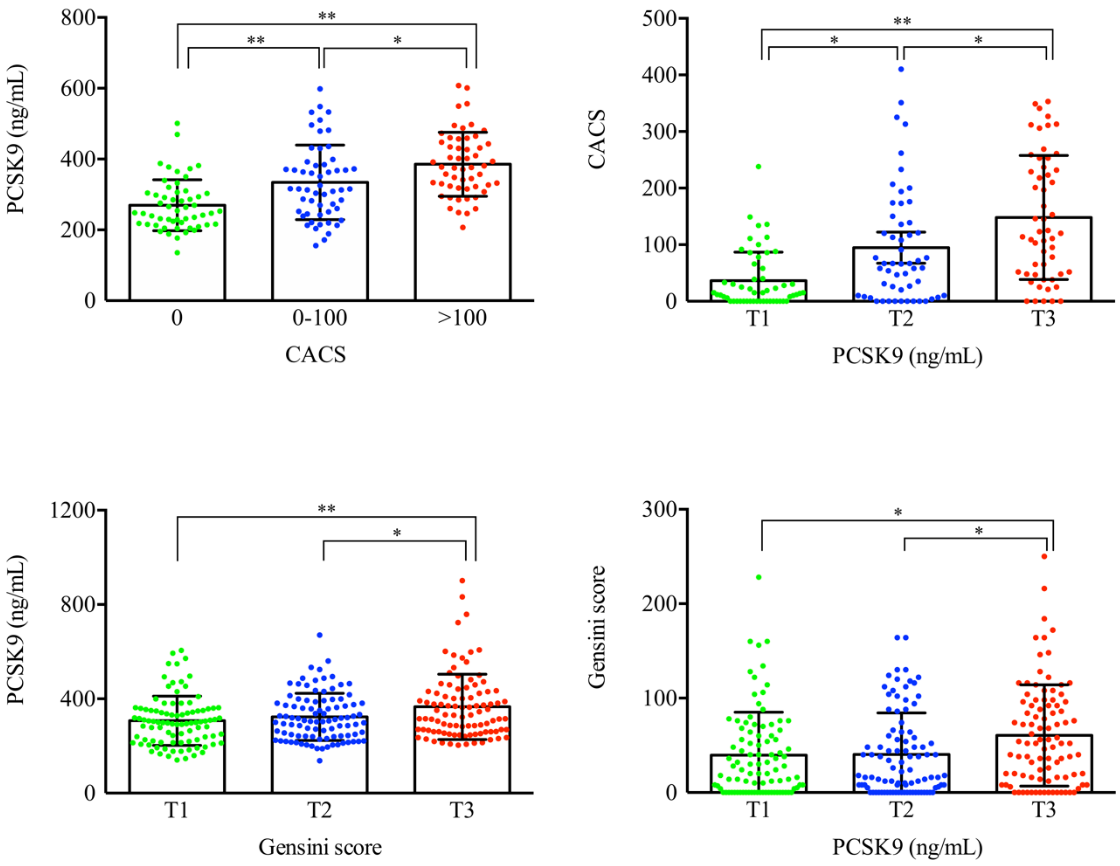

Note: * p <0.05; ** p<0.005

Figure S4. Receiver operating characteristic curve.

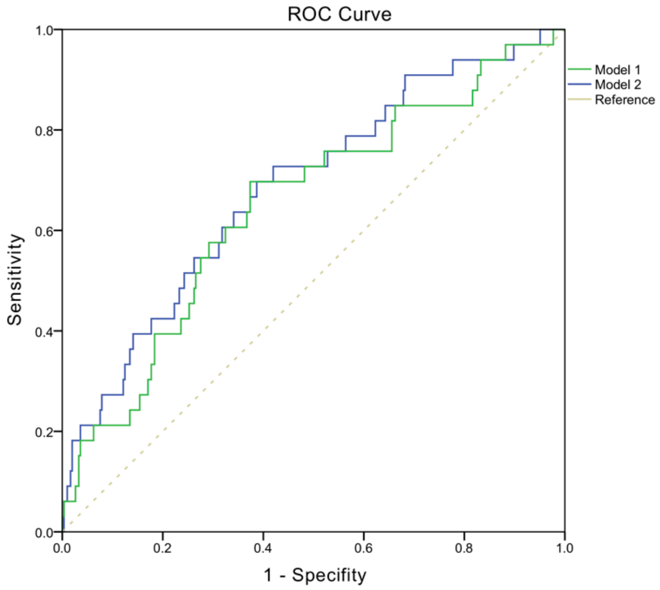

Model 1: including age, male, smoking, diabetes, LDL-C, HDL-C and Lp(a); AUC: 0.652 (95%CI: 0.549-0.754)

Model 2: Model 1+ PCSK9; AUC: 0.682 (95%CI: 0.583 - 0.780)
